# Addressing Stem Cell Therapeutic Approaches in Pathobiology of Diabetes and Its Complications

**DOI:** 10.1155/2018/7806435

**Published:** 2018-06-25

**Authors:** Bou-Yue Peng, Navneet Kumar Dubey, Viraj Krishna Mishra, Feng-Chou Tsai, Rajni Dubey, Win-Ping Deng, Hong-Jian Wei

**Affiliations:** ^1^School of Dentistry, College of Oral Medicine, Taipei Medical University, Taipei City 110, Taiwan; ^2^Department of Dentistry, Taipei Medical University Hospital, Taipei City 110, Taiwan; ^3^Ceramics and Biomaterials Research Group, Advanced Institute of Materials Science, Ton Duc Thang University, Ho Chi Minh City, Vietnam; ^4^Faculty of Applied Sciences, Ton Duc Thang University, Ho Chi Minh City, Vietnam; ^5^Applied Biotech Engineering Centre (ABEC), Department of Biotechnology, Ambala College of Engineering and Applied Research, Ambala, India; ^6^Department of Stem Cell Research, Cosmetic Clinic Group, Taipei City 110, Taiwan; ^7^Graduate Institute of Food Science and Technology, National Taiwan University, Taipei City 106, Taiwan; ^8^Stem Cell Research Center, College of Oral Medicine, Taipei Medical University, Taipei, Taiwan; ^9^Graduate Institute of Basic Medicine, Fu Jen Catholic University, New Taipei City 242, Taiwan; ^10^School of Dental Technology, College of Oral Medicine, Taipei Medical University, Taipei City 110, Taiwan

## Abstract

High morbidity and mortality of diabetes mellitus (DM) throughout the human population is a serious threat which needs to be addressed cautiously. Type 1 diabetes mellitus (T1DM) and type 2 diabetes mellitus (T2DM) are most prevalent forms. Disruption in insulin regulation and resistance leads to increased formation and accumulation of advanced end products (AGEs), which further enhance oxidative and nitrosative stress leading to microvascular (retinopathy, neuropathy, and nephropathy) and macrovascular complications. These complications affect the normal function of organ and tissues and may cause life-threatening disorders, if hyperglycemia persists and improperly controlled. Current and traditional treatment procedures are only focused on to regulate the insulin level and do not cure the diabetic complications. Pancreatic transplantation seemed a viable alternative; however, it is limited due to lack of donors. Cell-based therapy such as stem cells is considered as a promising therapeutic agent against DM and diabetic complications owing to their multilineage differentiation and regeneration potential. Previous studies have demonstrated the various impacts of both pluripotent and multipotent stem cells on DM and its micro- and macrovascular complications. Therefore, this review summarizes the potential of stem cells to treat DM and its related complications.

## 1. Introduction

The diabetes mellitus (DM), one of the most prevalent noncommunicable disease, is characterized by hyperglycemia leading to the development of severe life-threatening complications [1, 2]. Recent decades have witnessed a sudden increase of diabetes throughout the world, in spite of numerous efforts made to control to outspread of this metabolic disorder. Currently, type 1 diabetes mellitus (T1DM) and type 2 diabetes mellitus (T2DM) are the most prevalent type of diabetes. The T1DM, which is also known as insulin-dependent DM, is caused due to impairment in regulation of blood glucose by absolute destruction of insulin-producing *β*-cells, whereas insufficient or no response to insulin is attributed to the pathogenesis of T2DM. The International Diabetes Federation (IDF) reported that the number of diabetic population will increase from 415 million in 2015 to 642 million by 2040 [2]. Of note, any defect in insulin regulation in blood triggers the in metabolic disorders of carbohydrate, fat, and protein leading to a condition of hyperglycemia [3]. Insulin secretion is mainly stimulated by glucose; however, other factors such as amino acids, fatty acids, acetylcholine, pituitary adenylate cyclase-activating polypeptide (PACAP), glucose-dependent insulinotropic polypeptide (GIP), and glucagon-like peptide-1 (GLP-1) also participate in regulating the metabolism of their respective biomolecules [4]. The thirst, polydipsia, weight loss, polyuria, and blurred vision are some common symptoms of diabetes; in severe cases, hyperglycemia along with ketoacidosis or nonketotic hyperosmolar conditions are prevalent [4].

Currently, diabetic retinopathy, nephropathy, and neuropathy are the major reported complications. The other complication also includes foot ulcer [3, 5]. These complications have been reported to mediate via advanced glycations end products (AGEs), which mainly are the posttranscriptional modified proteins or lipids, and might be excessively synthesized during hyperglycemic conditions or present in the diet. These high levels of AGE also disrupt the defense mechanisms and assist in the destruction of *β*-cells [6]. 1Specifically, AGEs bind to their multiligands, known as a receptor of advanced glycation end products (RAGE), which activates different kinase and NADPH oxidase leading increased levels of ROS and further promotes the synthesis of more AGEs, thereby triggering cell-damaging mechanisms [7–9]. Notably, the AGEs not only destroy insulin-producing cells but also develop insulin resistance, a major symptom of T2DM [10].

It is well-known that the exercise and diet control are helpful to manage glucose level at initial stage [11]. The use of therapeutic insulin and other external hypoglycemic agents have also been employed to control the glucose level in blood, yet they are not capable enough to mimic the natural activity of endogenous insulin and may result in a hypoglycemic coma [12, 13]. The other therapeutic approach is transplantation of pancreas or islet cells; however, this approach is limited due to the lack of donors and surgical and postsurgical complexities associated with therapy [14].

**Figure 1:**
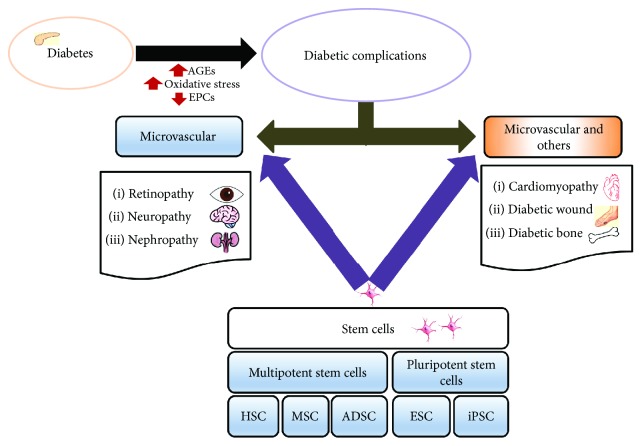
Schematic overview of stem cell therapy in diabetic complications. AGEs: advanced glycated end products; EPCs: epithelial progenitor cells; MSC: mesenchymal stromal cells; HSCs: hematopoietic stem cells; ADSC: adipose-derived stem cells; ESCs: embryonic stem cells; iPSCs: induced pluripotent stem cells.

In general, stem cell is a population of cells defined by its ability to indefinitely expand, self-renew, and undergo asymmetric divisions to produce progeny cells committed to specific differentiation lineages [15]. Embryonic stem cells, a pluripotent cell derived from the inner cell mass of a blastocyst, are capable of generating almost every cell types of the body but are unable to form an entire organism. Multipotent stem cells reside within various niches in the body and are limited to differentiating into specialized cell types of their tissue of origin such as mesenchymal stem cells and hematopoietic stem cells [16]. Stem cells are important for living organisms due to their functions of homeostatic tissue maintenance and replacing dysfunctional and senescent cells. Given their remarkable regenerative capacities, stem cells are being applied in treatments for various diseases as a novel potential therapeutic intervention, which is also referred to as regenerative medicine (Figure 1). In previous years, the role of stem cells has been extensively studied for their therapeutic potential to treat diabetic pathology and related complications. Therefore, this article reviewed the possibilities of stem cell therapies in diabetes and its associated complications.

## 2. Stem Cells in Treatment of Diabetes

**Figure 2:**
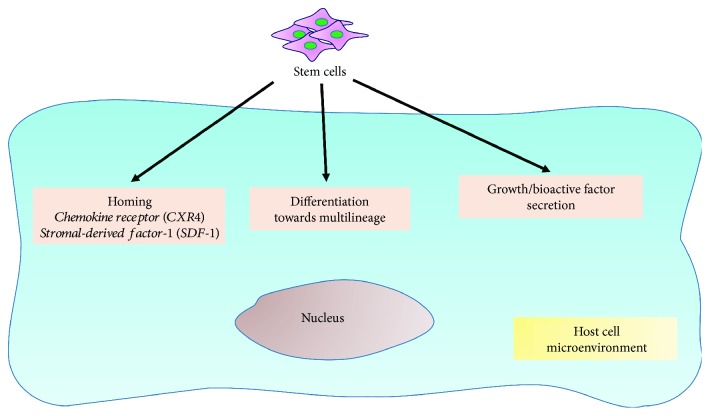
The possible mechanistic insight of therapeutic action of stem cells. During repair and regeneration, the transplanted MSC exhibit three modes of action, including homing, multilineage differentiation, and secretion of growth/bioactive factors.

Transplantation of insulin-producing cells [17] has paved the path to stem cell-based regeneration of insulin-secreting pancreatic *β*-cells [18]. Stem cells are unspecialized having the potential to regenerate and differentiate into specialized cells such as myocyte, hepatocyte, leukocyte, lymphocytes, erythrocytes, muscles, and nerve cells under proper environmental condition and signal [19]. On the basis of cell source, stem cells are generally classified as embryonic stem cells (ESCs) or adult stem cells (ASC). However, stem cells are also classified on the basis of origin, potential methods of derivations, and so on [19]. ESCs or pluripotent stem cells are isolated from inner cell mass of the blastocyst and have the potential to differentiate in different germ cell lines. However, the ethical issues make it very difficult to explore its potential to regenerate insulin-secreting cells. Notably, ASCs are multipotent stem cells and have the capacity to differentiate into only fewer cell types [17, 19]. ASC such as hematopoietic stem cell (HSC) not only multiply itself but also develop into blood cells, whereas mesenchymal stem cells (MSCs) trigger the generation of fat, bone, and cartilage. ASC also helps in repair and replacement of damaged tissues along with developments of the central nervous system and muscle cells. The therapeutic potential of stem cells may be ascribed to three major embodied mechanisms of action (Figure 2). First, the systemically administered stem cells undergo “homing” which further migrate to the site of injury possibly due to chemoattraction mediated by cell surface receptors such as the chemokine receptors. Although the exact mechanism of stem cells and endothelial interaction at the target site is not well established, the integrins and selectins have been suggested to mediate such interactions [20, 21]. The stem cell transmigration to the focal point of injury occurs across the endothelium through vascular cell adhesion molecule 1 (VCAM-1) and G-protein-coupled receptor signaling [22]. Secondly, the transplanted stem cell may undergo differentiation into multiple cell types, which after local engraftment can replace damaged tissues and induce restoration of their function [23, 24]. Thirdly, stem cell may also secrete growth/bioactive factors, which may potentially positively influence both local as well as systemic physiological processes [25].

## 3. Stem Cell-Derived Secretome in Organ Repair and Regeneration

**Figure 3:**
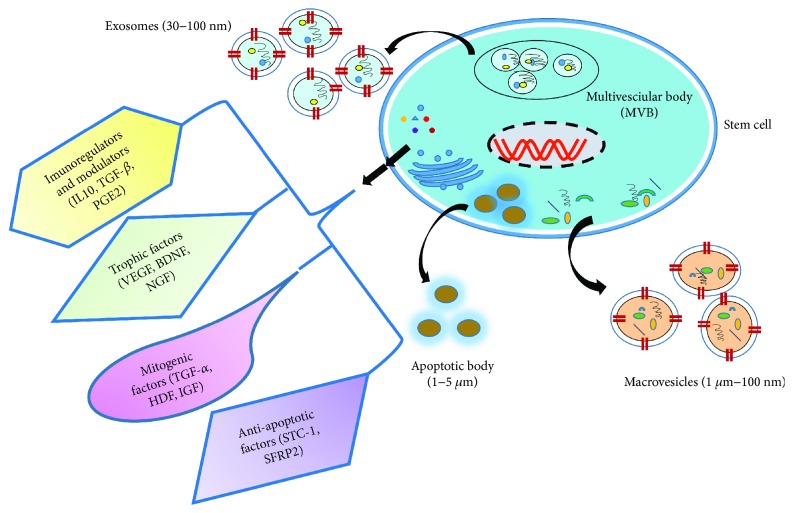
Mesenchymal stem cell-derived secretome and extracellular vesicles. IL: interleukin; TGF-*β*: transforming growth factor beta; PGE2: prostaglandins E2; VEGF: vascular endothelial growth factor; BDNF: brain-derived neurotrophic factor; NGF: nerve growth factor; HGF: hepatocyte growth factor; IGF: insulin-derived growth factor; STC-1: stanniocalcin-1; SFRP2: secreted frizzled-related protein 2.

Regeneration and repair activities of stem cells depend on their differentiation potential to replace the damaged or injured tissues [26]. Recent in vivo studies have established the fact that most of the transplanted MSCs are cleared rapidly from the in vivo microenvironment, thus limiting the regenerative therapeutic potential of stem cell differentiation to direct organ repair [20]. Therefore, their paracrine and immunomodulatory function of MSCs seems more effective through cellular communication without physical contact between cells, along with secreted trophic factors, extracellular RNAs, and miRNA which leads to cellular modulation, thereby triggering change in the microenvironment [21]. Various studies have documented the role of secretory factors of MSCs in tissue repair and regeneration via regulating inflammatory and allogenic immune response [23–25, 27]. It is clearly evident from recent reports that MSCs release soluble paracrine factors which regulate cellular proliferation, migration, differentiation, immunomodulation, and anti-inflammatory response through p38 MAPK, Akt, STAT-3, and TNF receptor pathways [28]. Stem cell-specific secretome includes the extracellular molecules such as extracellular vesicles (EVs), soluble proteins (e.g., chemokines, cytokines, and growth factors), lipids, and free nucleic acids [29, 30]. These EVs are produced by internal budding and when released into cellular microenvironment promotes regeneration of injured/damaged cells similar to stem cells after endocytosis; this regeneration procedure is mediated by receptor-ligand interaction, fusion or transfer of proteins, and nucleic acids or miRNA [31–34]. Based on their physical characteristics, EVs are further categorized among exosomes, apoptotic bodies, and microvesicles (MVs) (Figure 3) [35]. Exosomes are made up of spherical bi-lipid layer ranging from 30–100 nm in size. These membrane vesicles are released by various cells and considered as critical component for cellular communications, and in altering cellular signaling has rendered it an interesting candidate in regenerative therapy [36]. Exosomes promote specific interaction with targeted tissues/cells along with the disposal of unwanted proteins, antigen presentation, genetic exchange, immune responses, angiogenesis, inflammation, tumor metastasis, and spreading of pathogens or oncogenes [28, 37, 38]. Furthermore, apoptotic bodies are released from cells undergoing programmed death as blebs of 1–5 *μ*m in diameter [39]. Besides these secretomes, the expression of factors such as vascular endothelial growth factor (VEGF), fibroblast growth factor-2 (FGF-2), hepatocyte growth factor (HGF), insulin-derived growth factor-1 (IGF-1), and thymosin B4 (TB4) is also released and is regulated by Akt signaling [40]. Interestingly, the increase in expression level of these factors has been observed under hypoxic conditioned medium. The increased production of VEGF in MSCs under normoxia or hypoxia has been found to be associated with STAT3 and p38 MAPK signaling pathways [28, 41], whereas in adult rat bone marrow multipotent progenitor cells (rMAPCs), JAK2/STAT3 signaling pathways have been ascribed [42]. Moreover, another study suggests that transforming growth factor-*α*- (TGF-*α*-) induced VEGF production is associated with MEK and PI3-K signaling pathways in homogenous human BM-MSCs [43]. These observations indicate the varying signaling pathways are associated with VEGF production in different species [28]. Besides, the expression of TGF-*β*1 in rat MAPCs has also been linked with STAT3 pathway [42]. TNF receptor (TNFR) and associated signaling pathways also plays a critical role in expression of paracrine factors such as VEGF, TNF, cytokines, and IL-6 [28, 44]. It has also been reported that the production of HGF in human MSCs is closely associated with TNF and TGF-*α*/epidermal growth factor (EGF) receptors and MEK, p38, and PI-3K signaling pathways [45], where the TNF receptor 1 played role in decrease of HGF, when stimulated with TGF-*α* and TNF-*α*. A comparative study of paracrine factor profile of swine and human bone marrow MSCs showed that both cell cultures produced similar factors including VEGF and endothelin, along with other different paracrine factors under various conditions, which indicate that secretion of paracrine factors varies according to the species [28, 46]. Apart from this, the age also impacts proliferation rate of MSCs and their secretome level of paracrine factors. In a recent study, p38 and ERK signaling pathways seemed to be associated with cytokine and growth factors in neonatal BM-MSCs [47]. Along with the abovementioned factors, the gender [48, 49], disease status [50, 51], and environmental factors also significantly influence the type and level of secretory factors of MSCs [28].

Homeostasis, cell development, and cell repair/regeneration/survival are mediated by membrane protein and cell adhesion biomolecules (integrins, tetraspanins, and cadherins) which direct receptor-mediated cellular communication [52, 53], whereas coupling of cellular cytoplasm is mediated by gap junctions [21, 54, 55]. Stem cells lack gap junction; however, differentiated cells may communicate through gap junctions. This was evidenced in a report in which BM-MSCs were able to differentiate into cardiac cells via their communication to near myocytes through gap junction [55, 56]. Besides, tunneling nanotubes (TNTs) are a newly explored actin-based elements involved in long distance-based cellular communication [57, 58], leading to tissue developments and regeneration [21, 59].

## 4. Stem Cells Therapy in T1DM

Insulin-secreting *β*-cells become nonfunctional in T1DM, and this condition primarily arises due to autoimmune destruction of cells causing hyperglycemia. Traditional insulin therapy assists to control blood glucose level; however, it has proven ineffective in the long-term. Islet transplantation therapy is limited due to the availability of pancreatic cells, cell rejection, use of immunosuppressive drugs, and other complexities [17, 60]. These limitations could be avoided through stem cell therapies, owing to their very low immunogenic potential, immune-privileged, and immunomodulating properties [61–66]. Stem cells are also prone to genetic modification, through which the desired MHC complex may be introduced to control chance of immune rejections [67]. Furthermore, MSC has also been reported for their role in inhibition of T-cell proliferation, development of dendritic cells (DCs), and B-cell proliferation [63, 64, 68]. These reports are indicative of the immunosuppressive role of stem cells in transplantation therapy; however, more studies are required to establish their clinical significance.

In recent years, stem cells are emerging as a potential candidate for efficacious treatment for T1DM as these cells are capable to differentiate into mature *β*-cells in presence of required signals [12, 69]. The immunomodulation properties of stem cells can be helpful to control a balance between *β*-cell destruction and their regeneration [70]. Mouse ESCs (mESCs) have been widely studied and reported to promote the differentiation of insulin-producing cells under induced conditions to avoid ethical conflicts. ESC controls self-renewal by regulating the expression of different transcription factors such as Oct4, Sox2, and Nanog in presence of suitable medium [71]; germ cell nuclear factor (GCNF) and phosphoinositide kinase inhibitors catalyze the differentiation of specific functional cells. The designed media and transcription factors (Pax4 or Pdx-1) are reported for their potential to generate insulin-secreting cells [71–75]. Human ESC (hESC) has been demonstrated to differentiate into functional *β*-cells in vivo [76]. However, the regulation of differentiation, teratoma formation, risk of viral infection, transplantation rejection, and ethical issues are still major bottlenecks to utilize it as a potential therapy.

iPSCs are the new alternatives of ESCs to avoid ethical concerns. iPSCs are mainly somatic cells which are reprogrammed to pluripotency. Though the traditional method of generating iPSCs are controversial, the iPSCs developed by Takahashi and Yamanaka have accelerated their use for generation of functional cells; in particular, the mouse and human fibroblasts have already been reprogrammed into pluripotent cells by using Oct3/4, Sox2, c-MYC/Lin28, and Nanog/Klf4 transcription factors [77, 78]. Miyazaki et al. also reprogrammed cancerous cells into induced pluripotent cells using the same transcription factors [79]. Kim et al. suggested that somatic cells which express any of the transcription factors required for induction of pluripotency will reduce the requirement of complete transcription factors [80]. For insulin regulation, mouse fibroblast cells have also been induced into pluripotent stem cells, which were further triggered to differentiate into insulin-producing cells for insulin regulation [81]. The potential of iPSCs in diabetes treatment is promising; however, the chances of tumor formation and immune response to transplantation need to be critically evaluated [70].

Adult stem cells such as hepatic stem cells, bone marrow-hematopoietic stem cells (BM-HSCs), and mesenchymal stromal cells (MSCs) derived from the bone marrow and umbilical cord blood (UCB) and adipose tissue-derived MSCs (ADSCs) have been explored for their potential to generate insulin-producing cells. The endodermal nature of pancreatic cells makes hepatic stem cells a prospective stem cell source for therapeutic use. In various studies study, Pdx-1 was used to induce growth of *β*-cell precursors from hepatic tissues [69, 72, 82, 83]. Mouse and human hepatic stem cells were differentiated into insulin-secreting *β*-like cells and used to overcome the condition of hyperglycemia [84]. The application of hepatic stem cells to induce the regeneration of insulin-producing cells is promising; however, further extensive research is required to establish the protocols for clinical application. Since MSCs have the potential to differentiate into pancreatic cells as well as to heal damaged cells, these have been exploited in treatment of T1DM [85]. BM-MSCs are also able to promote graft acceptance and reduce autoimmunity [70, 86–88]. However, BM-MSCs' potential for stem cell therapy is limited by lack of standardized methods, difficulty in in vivo differentiation, and the possibility of tumor induction [70]. ADSCs are closely similar to the BMSCs and clinically accepted for their therapeutic potential due to ease of isolation with abundant cell numbers. The ADSCs have also been successfully used to counter type 1 diabetes in mice, and its potential to counteract the graft rejection response enhances the chance of success of T1DM therapy [70, 89–91].

## 5. Stem Cell Therapy in T2DM

Insulin resistance and a decrease in insulin production are the characteristics of T2DM. Conventional treatment approach includes using external insulin and use of oral antidiabetic drugs [92]. However, the regular use of in vitro insulin makes T2DM patients insulin resistant and contemporary therapy does not address this complication [93]. Transplantation of islet cells was once considered as a promising therapeutic approach; however, this approach is not common due to lack of donors, ethical conflict, and risk of immunogenicity. Regeneration and multipotent potential of stem cells make it an integral candidate for cell-based therapy. Stem cells such as BMSCs, ADSCs, ESCs, and iPSCs are able to differentiate into insulin-producing cells resulting in an increase in insulin level in patients under defined conditions and well-established procedures [94, 95]. Intrapancreatic autologous stem cell injection under hyperbaric oxygen condition regulates glycemic condition and insulin level [96]. Similar results were also reported when autologous bone marrow-derived stem cells were intra-arterially injected [97]. MSCs have improved islet function and controlled insulin resistance in T2DM. Various trials are under clinical phase I and II, however, only a few of them are based on random and placebo-controlled [92]. Moreover, the establishment of the exact pathway in stem cell-based treatment of T2DM still needs to be well established.

## 6. Stem Cells in Diabetic Complications

Diabetes not only disrupts the blood glucose regulations but also alters the metabolism in long run if poorly managed. As a result, micro- and macrovascular complications occur [98–100]. The microvascular complications arise due to impairment in small blood vessels under chronic hyperglycemic milieu. Some of these complications are diabetic retinopathy, neuropathy, and nephropathy, whereas the macrovascular complication is caused by damage to arteries leading to cardiovascular disease (CVD), coronary artery disease (CAD), peripheral arterial disease, myocardial infarction (MI), and stroke. Diabetes-associated disorders like osteoporosis, osteoarthritis, foot ulcers, and diabetic cardiomyopathy are some other secondary complications [101–104]. Regeneration and differentiation capability of stem cells make it possible to explore their therapeutic potential to treat and control diabetic complications. Specifically, the multipotent stem cells such as MSCs/HSCs, progenitor stem cells, tissue-specific stem cells, and pluripotent stem cells (ESCs and iPSCs) are considered to counter the diabetes-associated disorders [98, 100]. Therefore, the selection of the suitable source of stem cells is critical to ensure the differentiation of stem cells into both endothelial and perivascular cells to repair diabetic complications [105]. In the further sections, we have discussed the role of stem cell therapy in several diabetic complications.

## 7. Microvascular Diabetic Complication and Stem Cells

### 7.1. Stem Cells and Diabetic Retinopathy

Abnormal ocular vascularity and retinal lesions lead to the development of blindness in retinopathy. The diabetic retinopathy (DR) is more prevalent in T1DM patients; however, it is hard to differentiate its incidence between T1DM and T2DM [106, 107]. DR is classed as either nonproliferative diabetic retinopathy (NPDR) or as proliferative diabetic retinopathy (PDR) [108]. Microvascular alterations cause retinal ischemia in NPDR, whereas PDR is caused by disruption of the ocular vitreous cavity due to the generation of abnormal blood cells leading to blindness [106, 109, 110]. Contemporary therapies such as vitrectomy and laser photocoagulation do not address the root cause of the disease [111]. Thus, stem cells seem as the most effective long-term treatment option for DR. In previous studies, MSCs and HSCs have been reported for their potential to differentiate into ocular cells to repair retinal damages [104]. In a seminal study in a rat model, it has been evidenced that MSCs are capable enough to mitigate and recover the loss of visual impairments [112, 113]. Scalinci et al. found that neuroprotective growth factors such as brain-derived neurotrophic factor (BDNF), ciliary-derived neurotrophic factor (CTNF), nerve growth factor (NGF), glial-derived neurotrophic factor (GDNF), and basic fibroblast growth factor (bFGF) were significantly increased in DR rata injected with hMSCs [114]. However, inferior homing capacity of intravitreally administered MSCs and increased level of vascular endothelial growth factor (VEGF), a factor responsible for vascular lesion, were found. In another study, atorvastatin, a reductase inhibitor enzyme, had also reduced VEGF when MSCs were injected and hypoxic condition was maintained subsequently [115]. Siqueira et al. also demonstrated that BM-HSCs led to an improved visual activity [116]. Further, in animal models, the injected EPCs derived from murine BMSCs and hUCB promoted neovascularization and ameliorated DR [117–119].

### 7.2. Stem Cell in Diabetic Neuropathy

Diabetic neuropathy (DN) is one of the most prevalent complications among T1DM and T2DM patients, which may lead to foot ulcers and limb amputation [120]. DN becomes more chronic with an increase in the level of hyperglycemia and with the passage of time [121, 122]. Microvascular factors, metabolic regulations, unregulated glucose level, increased glycated hemoglobin level, oxidative and nitrosative stress, and reduced blood flow rate (due to the accumulation of ROS) are some factors which are attributed to the incidence of DN [121, 123]. ROS and reactive nitrogen species reduce blood flow leading to microvascular ischemia, which finally disrupts the function of the nerve [124]. Prolonged hyperglycemia also promotes the production of AGEs which after binding to RAGEs trigger an inflammatory response and enhance oxidative stress, leading to degeneration of Schwann cells. These cells not only insulate neuron but also regulate nerve regeneration, and any oxidation-mediated loss in their function promotes DN among diabetic patients [124, 125].

To develop an efficient therapy against DN, the treatment procedure should address both neurotrophic and angiogenic requirements simultaneously. Considering these requirements, stem cells seem viable and efficient, as they are capable to synthesize neurotrophic, angiogenic, and other essential factors required for regeneration of neuronal and vascular cells. The multilineage potential and adherent nature of MSCs cells helps it to secrete factors which are essential for neurotrophic and angiogenic effects. Different studies have revealed that MSCs improved DN symptoms in streptozotocin- (STZ-) induced diabetic rats. Though this treatment, VEGF and fibroblast growth factor-2 (FGF2) were increased and the capillary number to muscle fiber ratio in soleus muscles and sural nerve morphometry were improved [126]. In a multiple intravenous MSC treatment in STZ-induced T2DM rats, a controlled hyperglycemia with enhanced serum insulin and C-peptide was found at 9 weeks [127]. Motor and sensory nerve function restored in BMSC-treated STZ-induced diabetic rat [128]. Nerve regeneration has also been demonstrated with combined treatment of human MSCs and poly (3-hydroxybutyrate-co-3-hydroxyhexanoate) in Sprague-Dawley albino rats [129]. These animal-based studies strongly indicate that MSCs should have essential elements to address DN complications. However, lack of established clinical procedures, risk of tumor formation, and lack of understanding of clear mechanism are posing challenges to MSCs' candidacy as a therapeutic agent for DN [120].

### 7.3. Stem Cells in Diabetic Nephropathy

Diabetic nephropathy (DNP) is responsible for high mortality and a major contributor in end-stage chronic renal disease [130, 131]. Podocytes, the matrix molecule-synthesizing elements in the glomerular basement membrane, are injured and lost in DNP, leading to proteinuria and fibrosis and finally to renal failure. The regeneration capacities of podocytes are limited when injured, and it will adversely affect the glomerular barrier, further aggravating proteinuria [132]. Proteinuria promotes the dysfunction of proximal tubular epithelial cells (PTECs) by increasing fibrosis and tubulointerstitial inflammation, resulting in decreased renal activity [133]. Increase in immune cells in the interstitium is a characteristic feature of DNP [131]. Prolong hyperglycemia, AGEs, and glycated albumin enhance the inflammatory and fibrotic properties of PTECs [134]. AGEs also activate the renin-angiotensin system (RAS), triggering the secretion of ROS thereby increasing the formation of cytokine and growth factors [135]. In an important study, an enhanced DNP symptom in mice was revealed through an increased level of carboxymethyl-lysine (CML) an advanced glycation end product [136]. However, the ESCs, under the presence of required growth factors, including retinoic acid, activin A, BMP-2, BMP-7, and FGF-7, can be differentiated into renal cells [137, 138]. Various studies have also successfully differentiated iPSCs into renal cells to improvise the DNP characteristics [139, 140]. MSCs have also been introduced into an STZ-induced diabetic rat to repair renal damage and regenerate insulin-secreting cells [141, 142], whereas the stromal cell-derived factor (SDF-1) promoted homing of MSCs when released in the kidneys [143]. Nagaishi et al. demonstrated that BM-MSCs inhibited the proinflammatory cytokine, TGF-*β*1, and fibrosis in tubular interstitium They further revealed exosome-assisted antiapoptotic effect in tight junction structures of tubular interstitial cells indicating improved DNP [130]. The MSCs also exerted regenerative and protective effects in DNP by improvement in fibrosis and glomerulosclerosis, possibly via reducing the loss of podocytes and increased the secretion of BMP-7 [144]. BM-MSC treatment has regulated the serum level of insulin, hemeoxygenase-1, AGEs, and glucose with recovery in renal function [145]. Overall, the role of MSCs in the treatment of DNP is prospective, however, it is limited due to previously discussed hurdles.

## 8. Stem Cells in Macrovascular and Other Complications

DM patients are prone to atherosclerosis in large arteries finally developing macrovascular complication in the artery. Prolong hyperglycemia and atherosclerosis enhance the risk of myocardial infarction, artery disease, and stroke [98, 146]. CD 133 and CD34 are potent markers of cardiovascular diseases (CVD), and reduction in EPCs is used as an indicator of peripheral artery disease (PAD) [147–149]. Vascular stem cells (VSCs) are capable to differentiate EPCs and are a potential target for treatment of diabetic macrovascular complications. Vascular progenitor cell isolated from human vascular smooth muscle cells under proper condition was able to grow into vascular networks [150]. In a report, Keats and Khan proposed a hypothesis to develop vascular network from CD133+ VSC due to its ability to differentiate into EPCs and MPCs [105]. Further, the interaction between AGEs and RAGEs plays a critical role in the development of macroangiopathy and macrovascular complications [105].

### 8.1. Stem Cells in Diabetic Cardiomyopathy

Diabetic cardiomyopathy (DCM) is mainly developed due to cellular apoptosis. DCM reduces tissue-specific stem cells, intensifies fibrosis, and decreases perfusion in the capillaries [151, 152]. This complication is characterized by the reduced activity of metalloproteases-2 (MMP-2), high collagen in specific tissue, and upregulated activity of apoptotic factor MMP-9 [98]. However, MSCs have also been implicated in regenerating myocardial cells for restoring normal function of the heart. Specifically, administration of BM-MSCs has shown to improve diabetic myocardium in the T1DM rat by reducing collagen level and activity of MMP-9 [153]. Other stem cells such as ESCs, iPSCs, and cardiac stem cells had also been explored to recover myocardial infarction in animal models [154–156].

Besides, MSCs also induce myogenesis and angiogenesis by releasing various angiogenic, mitogenic, and antiapoptotic factors, including vascular endothelial growth factor (VEGF), insulin-like growth factor-1 (IGF-1), adrenomedullin (AM), and hepatocyte growth factor (HGF) [20]. This was demonstrated using a rat model of DCM [20], wherein intravenously administered rat BM-MSCs improved cardiac function via differentiating into cardiomyocytes and improved myogenesis and angiogenesis. In addition, the activity of MMP-2 was significantly increased, while MMP-9 increased, which led to enhanced myocardial arteriolar density and reduced collagen volume. MSCs also promoted the secretion of Bcl-2, hypoxia-related HOM-1, HSP-20, stromal cell-derived growth factor, and VEGF under hypoxic condition and stimulated neovascularization and restored myocardial function [157–159]. Notably, the site of injection and cell load has also been considered as determinants for improvement in myocardial infarction during MSC therapy [160].

### 8.2. Stem Cells in Diabetic Bone

T1DM and T2DM both interfere with normal osteogenic pathways, resulting in elevated risk of bone fractures and reduced ability of fracture healing. Bone-associated complications, affecting osteoblasts and osteoclasts, are mainly attributed to increased levels of AGEs, inflammation, and ROS [161]. AGEs not only block the osteoblastic differentiation and formation of mineralized matrix but also promote apoptosis of osteoblast, leading to impaired bone formation [162, 163]. Interaction between blood vessels and bone cells promotes regeneration and repair of the bone, which is disrupted in a hyperglycemic microenvironment, thereby hindering the repair of bone fracture [164]. Increase in secretion of TNF-*α*, IFN-*γ*-inducible protein 10 (IP-10), IL-1*β*, IL-6, and high-sensitivity C-reactive protein (hsCRP) was also reported after bone fracture in T2DM patients [165]. Current grafting procedures for treatments are limited due to rejection, difficulty in integration, long-term relief, and cost [166]. To overcome these challenges, the tissue engineering approaches have been used in MSCs are considered as leading therapeutic candidates [164]. MSCs are capable to differentiate into osteoblasts and also secrete factors such as VEGF and BMP-4 to promote bone cell regeneration [167, 168]. Studies have also used immortalized BMSCs in osteoarthritic recovery [169]. These studies showed the potential of MSC therapy in bone-associated disorders. However, further studies are still needed to establish a definite role of MSCs in the treatment of these disorders. Furthermore, the role of pluripotent and other adult stem cells in regeneration and repair of bone is also needed to be extensively explored.

### 8.3. Stem Cells in Wound Healing

Persistent and long-term hyperglycemia disrupts the wound healing capacity of T1DM and T2DM patients leading to chronic wound [170] and increases the risk of opportunistic infections. This chronic condition is developed due to impaired angiogenesis, uncontrolled release of growth factors, and incoherence in the accumulation of collagen matrix [98]. The increased rate of apoptosis of EPC and their numbers among DM patients have already been observed [171]. Additionally, the high level of inflammatory cytokines like TNF-*α*, CRP, and IL-8 are also found to be associated with poor wound healing capacity. Other factors related to collagen metabolism such as keratinocyte growth factor (KGF), transforming growth factor *β* (TGF-*β*), epidermal growth factor (EGF), platelet-derived growth factor (PDGF), and VEGF are also associated with chronic diabetic wound [172]. However, studies have demonstrated that both the MSCs and EPCs were recruited at the injury site and exerted the healing effect [98]. In a study, the iPSCs showed wound healing in diabetic patients by increasing the level of proangiogenic factors and controlled the activity of protein kinase C delta (PKC-*δ*) [173]. Another study demonstrated increased collagen accumulation in diabetic fascial wounds of rats, when treated with BM-MSCs which have been ascribed to the secretome of growth factors such as TGF-*β*, KGF, EGF, PDGF, and VEGF, essential to healing efficacy [174]. These factors also improve cell adhesion and promote an increase in secretion of chemokines at wound site [157, 158]. In various previous studies on animal models, MSC therapy has already been evidenced with an improved wound healing, for which different mechanisms have been explained [104, 174–177].

## 9. Combinatorial and Coculture Approaches in Stem Cell-Based Therapy of Diabetes and Its Complications

Therapeutic potency of stem cells is still in developmental phase for diabetic treatment, and the interactive effect of other chemical molecules on stem cell-based therapy is needed to be widely screened to improve their efficacy and safety. The pathological state such as diabetic wound healing have limited therapeutic options; however, a therapeutic combinational approach using ADSCs and exendin-4 (Ex-4) significantly improved the wound healing than singleton treatment in diabetic mice [178]. This effect was exhibited through proliferation and migration of endothelial cells and keratinocytes. Another combinatorial effect of MSCs and obestatin significantly improved the pancreatic damage in the T2DM rat model [179]. This was achieved through obestatin-mediated promotion of proliferation of active *β*-cells or islet-like cell clusters in vitro. Similarly, a study demonstrated the cumulative therapeutic effect of icariin and MSCs towards diabetes-induced erectile dysfunction, where icariin enhanced the therapeutic potential of ADSCs through its antioxidative and antiapoptotic activities [180]. In an interesting study, murine ESCs differentiated rapidly into pancreatic *β*-cells by using activin A, all-trans retinoic acid and some other factors such as Matrigel [181]. These differentiated cells were able to control the blood glucose level in vivo in the diabetic murine model; however, tumor formation in the kidney limited the use of transplanted cells. Besides, the impaired endothelial progenitor cell (EPC) homing reduce the wound healing ability in the diabetic microenvironment, which is associated with reduced expression of stromal cell-derived factor-1*α* (SDF-1*α*). However, the homing of EPCs can be improved at wound site under hyperoxia and via administration of SDF-1*α* [182]. In a clinical study, the synergistic administration of hyperbaric oxygen and intrapancreatic autologous stem cell was effective in controlling the metabolic level of insulin in T2DM patients [96]. It has also been shown that the preconditioning of the stem cell might improve the efficacy of cell-based therapy. MSCs harvested from diabetic mice were preconditioned in presence of insulin-like growth factor-1 (IGF-1) and fibroblast growth factor-2 (FGF-2) in medium and were further acclimatized under hypoxia and high glucose condition. After implantation of conditioned MSCs, the improvement in heart condition of diabetic mice was observed, indicating stem cell-based strategies to treat diabetic heart failure [183].

Recently, coculture techniques have also been used to improvise the efficacy of stem cells through enhancing their differentiation potential. In a study, the ESCs were cocultured with hepatocytes and induced to differentiate into endodermal cells, which were further induced to differentiate into pancreatic islet cells in presence of Matrigel and retinoid [184]. Another experimental study showed that differentiated islet cell clusters from human Wharton's jelly-derived mesenchymal stem cells in the presence of rat pancreatic cells could suppress blood glucose level [185]. Cotransplantation of kidney-derived MSCs with islets in diabetic mice has also remodelled islet organization and vascularization and reduced hyperglycemia [186]. Similarly, a seminal study pointed out that the viability of isolated islet was improved, when cocultured with collagen mixed hydrogel (collagen type I, collagen type III, and laminin) [187]. It is of note that the coculture system is used not only in improving therapeutic efficacy of stem cells but also to contemplate the pathogenesis of diabetes. In a conclusive study, a coculture system of BMSCs and macrophage helped to understand that association between local inflammation and immune response promotes diabetic periodontitis, particularly by upregulating the expression of chemokine (C-C motif) ligand 2 (CCL2) and TNF-*α* in periodontal tissues [188].

## 10. Gene Editing in Stem Cell for Treatment of Diabetes and Its Complications

Recent developments in gene targeting, editing, and delivery have made it feasible to develop an effective and long-term therapy for the treatment of genetic disorders. Adult stem cells, such as HSCs and MSCs are considered as promising candidates for exploiting gene modification techniques in cell-based regenerative therapy [189–191]. Vectors derived from retroviruses and adenoviruses are most commonly used to transfer the genes in stem cells; however, the chances of random integration might be deleterious. The other limiting factor associated with gene editing is no retaining of the edited gene by stem cells during their ex vivo proliferation. To overcome the limitations of viral vectors genetic control elements such as scaffold attachment region (SAR) and chicken beta-globin locus are added into the vectors to effectively control the gene expression in stem cells [192]. In diabetic mice, the transplanted BM-MSC expressing pancreatic duodenal homeobox 1 (Pdx1) gene differentiated into insulin-releasing *β*-cell and controlled the glucose level [193]. Similarly, a seminal study showed that the transfected MSCs with vascular endothelial growth factor (VEGF) gene improved the erectile dysfunction in diabetic rats [194]. Though this stem cell-mediated gene therapy demonstrated successful results in rats, it possesses a few limitations as it was carried out only in the T1DM animal model and used adenovirus vector is not considered as a robust gene expression system. In a recent interesting study, the genetically modified human urine-derived stem cells with FGF2 gene significantly improved ED in T2DM SD-rat model [195].

Recent gene editing techniques such as zinc-finger nucleases (ZFNs), transcription activator-like effector nucleases (TALENs), and the clustered regularly interspaced short palindromic repeats-associated Cas protein system (CRISPR/Cas) seems promising to understand the role of specific genes in beta cell development and to manipulate the stem cell differentiation into insulin-producing cells [196]. The CRISPR/Cas9 system is currently favoured due to its modularity, flexibility, specificity, reduced toxicity, ease of designing target single-guide RNA (sgRNA) and reduced side effects. Gene-editing techniques have clearly established the role of transcription factor, neurogenin 3 in development of endocrine cells of pancreas, and demonstrated that even low expression of this factor is sufficient to promote the stem cell differentiation into insulin-producing beta cells [197]. Further, the CRISPR/Cas9 mediated deletion of CDKAL1, KCNJ11, and KCNQ1 genes in hESCs disrupted the regulated production of insulin in differentiated beta cells. These recent studies imply that human pluripotent stem cells can be exploited as an effective model to understand molecular development of insulin-producing pancreatic beta cells [196]. Furthermore, the clear understanding of genetic regulation will help in developing and controlling the differentiation of functional beta cells. Notably, gene editing in stem cells also help to escape immune response during transplantation of differentiated cells. This was evidenced in a study in which complete knock out of human leukocyte antigens (HLAs) class-I through disrupting beta 2-microglobulin (*β*2m) in hESCs maintained the cellular pluripotency level with significantly reduced immunogenicity [198].

## 11. Conclusions

The diabetic complications are the most prominent reason for high mortality among diabetic patients; therefore, due to proven repair and regeneration potential, the cell-based therapies, including pluripotent and multipotent adult stem cells are currently being considered. This therapeutic approach will not only be helpful to overcome the limitations of contemporary therapy but also provide a long-term cure for diabetes and its complications. However, extensive studies are needed to establish standard procedures for stem cell treatment in diabetic complications.
